# Supplementary material for: Metabolic alteration in oxylipins and endocannabinoids point to an important role for soluble epoxide hydrolase and inflammation in Alzheimer’s disease—finding from Alzheimer’s Disease Neuroimaging Initiative
Source: Alzheimers Res Ther. 2026 Jan 7;18:21. doi: 10.1186/s13195-025-01939-9 (PMC12857118; doi:10.1186/s13195-025-01939-9)
Supplement: Supplementary file 1 — Supplementary Material 1. [file 13195_2025_1939_MOESM1_ESM.pdf]

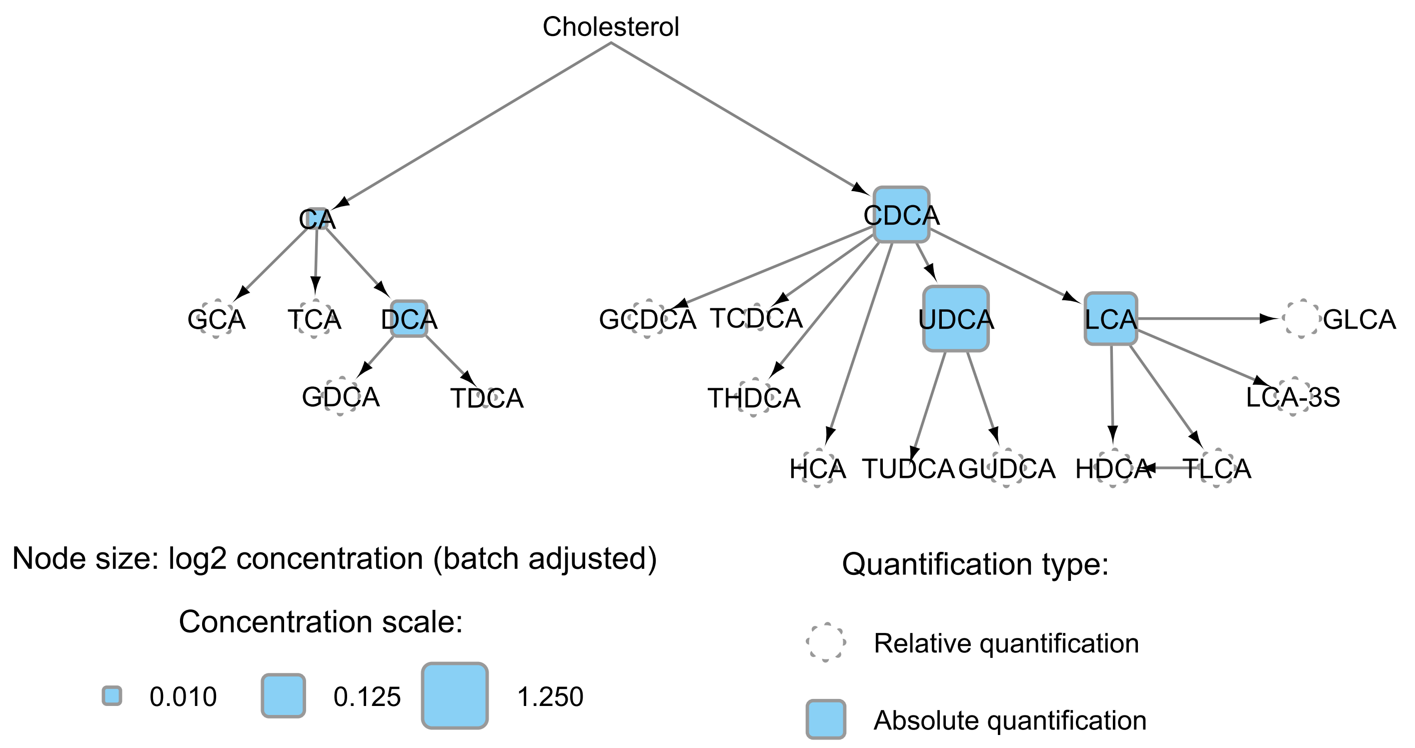

Supplemental Figure S1. Quantified bile acids projected onto their metabolic pathway with node sizes indicating their average concentration in samples.
