# Supplementary material for: Metabolic alteration in oxylipins and endocannabinoids point to an important role for soluble epoxide hydrolase and inflammation in Alzheimer’s disease—finding from Alzheimer’s Disease Neuroimaging Initiative
Source: Alzheimers Res Ther. 2026 Jan 7;18:21. doi: 10.1186/s13195-025-01939-9 (PMC12857118; doi:10.1186/s13195-025-01939-9)
Supplement: Supplementary file 3 — Supplementary Material 3. [file 13195_2025_1939_MOESM3_ESM.pdf]

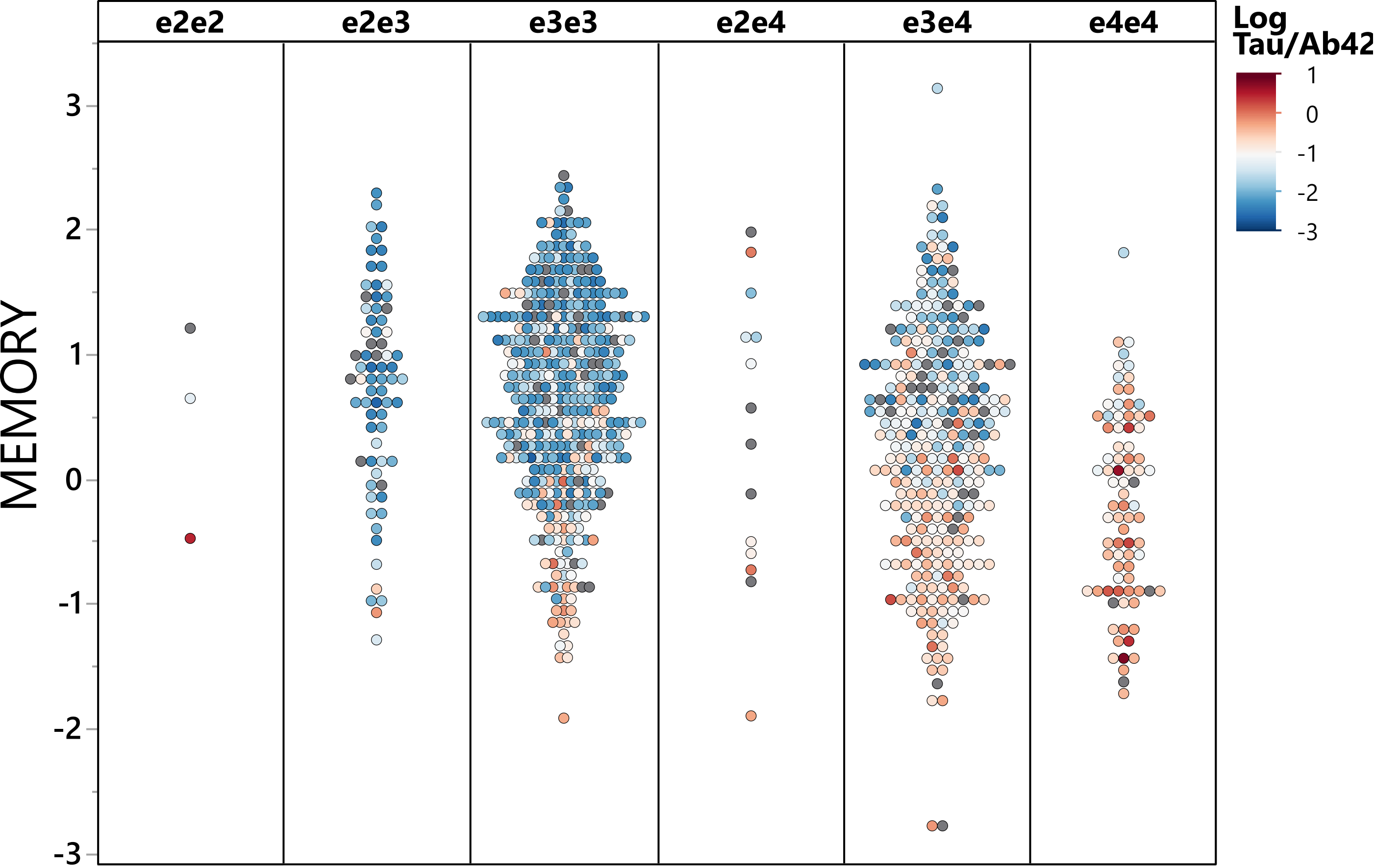

Supplemental Figure S3. Relation between Tau and Ab42 CSF biomarkers, the memory score and APOE genotype in selected ADNI2 subjects.
