# Supplementary material for: Metabolic alteration in oxylipins and endocannabinoids point to an important role for soluble epoxide hydrolase and inflammation in Alzheimer’s disease—finding from Alzheimer’s Disease Neuroimaging Initiative
Source: Alzheimers Res Ther. 2026 Jan 7;18:21. doi: 10.1186/s13195-025-01939-9 (PMC12857118; doi:10.1186/s13195-025-01939-9)
Supplement: Supplementary file 4 — Supplementary Material 4. [file 13195_2025_1939_MOESM4_ESM.pdf]

Table S1. Key biometrics stratified by sex

	Males								Females							
Y	Mean 1 AD	Std Dev 1 AD	Mean 1 CN-CN	Std Dev 1 CN-CN	Mean 1 MCI-AD	Std Dev 1 MCI-AD	Mean 1 MCI-MCI	Std Dev 1 MCI-MCI	Mean 2 AD	Std Dev 2 AD	Mean 2 CN-CN	Std Dev 2 CN-CN	Mean 2 MCI-AD	Std Dev 2 MCI-AD	Mean 2 MCI-MCI	Std Dev 2 MCI-MCI
Age	74.94	8.14	73.38	6.57	73.15	6.57	71.79	7.23	71.81	7.90	70.79	5.19	71.45	7.77	70.25	7.64
Baseline ADNI MEM	-0.90	0.46	1.08	0.65	-0.15	0.56	0.36	0.57	-0.85	0.62	1.25	0.47	-0.20	0.58	0.70	0.64
Baseline BMI	26.51	4.40	27.65	3.81	27.62	3.86	28.04	4.66	26.15	6.91	27.62	5.33	27.78	7.13	27.31	5.28
Education	16.46	2.64	17.29	2.36	16.47	2.77	16.42	2.63	14.85	2.56	16.24	2.37	15.59	2.43	15.40	2.64
