# Supplementary material for: Metabolic alteration in oxylipins and endocannabinoids point to an important role for soluble epoxide hydrolase and inflammation in Alzheimer’s disease—finding from Alzheimer’s Disease Neuroimaging Initiative
Source: Alzheimers Res Ther. 2026 Jan 7;18:21. doi: 10.1186/s13195-025-01939-9 (PMC12857118; doi:10.1186/s13195-025-01939-9)
Supplement: Supplementary file 5 — Supplementary Material 5. [file 13195_2025_1939_MOESM5_ESM.pdf]

Cluster	Metabolites	RSquare with Own Cluster	RSquare with Next Closest	1-RSquare Ratio	Anova p value female	Anova p value male	DunnettTest-MCI to HC female	DunnettTest-MCI to HC male	DunnettTest-AD to HC female	DunnettTest-AD to HC male
	sum_DiHETE	0.95	0.42	0.09						
	17_18_DiHETE	0.91	0.39	0.15						
1					0.00013	0.12	0.92	0.22	0.00028	0.073
	19_20_DiHDPA	0.75	0.32	0.36						
	14_15_DiHETE	0.75	0.4	0.41						
	GLCA/CA	0.83	0.3	0.24						
	GDCA/CA	0.83	0.36	0.27						
7	HDCA/CA	0.76	0.02	0.25	0.68	0.00096	0.66	0.04	0.67	0.00038
	UDCA/CA	0.74	0.02	0.26						
	GCA/CA	0.61	0.12	0.44						
	LCA_3S/CA	0.58	0.16	0.5						
	sum_DiHETrE	0.91	0.19	0.11						
	11_12_DiHETrE	0.87	0.2	0.16						
	14_15_DiHETrE	0.84	0.18	0.19						
2					0.00018	0.0056	0.95	0.0084	0.00042	0.0067
	8_9_DiHETrE	0.78	0.16	0.26						
	20_HETE	0.61	0.12	0.45						
	FA20.3_w6	0.35	0.12	0.74						
	LPA16.0	0.84	0.13	0.19						
	LPA20.3	0.78	0.2	0.27						
4					0.0039	0.38	0.0017	0.87	0.18	0.63
	LPA22.6	0.67	0.4	0.54						
	LPI16.0	0.3	0.03	0.73						
	19_20_DiHDPA/DHA	0.7	0.07	0.33						
	17_18_DiHETE/EPA	0.66	0.15	0.4						
5	14_15_DiHETE/EPA	0.58	0.14	0.49	2.7E-06	0.0051	0.61	0.95	0.00018	0.011
	19_20_DiHDPA/19_20_EpDPE	0.46	0.12	0.62						
	14,15-DiHETrE/AA	0.3	0.07	0.75						
	1_AG_2_AG	0.88	0.09	0.13						
	1_LG_2_LG	0.88	0.03	0.13						
6					0.48	0.0063	0.39	0.44	0.92	0.13
	GDCA	0.83	0.13	0.2						
	GLCA	0.83	0.17	0.21						
	sum_HDoHE	0.93	0.26	0.09						
3	17_HDoHE	0.69	0.26	0.42	0.065	0.36	0.037	0.26	0.43	0.54
	16_HDoHE	0.62	0.15	0.45						
	5_HEPE	0.85	0.37	0.23						
	8_HDoHE	0.79	0.4	0.34						
8					0.16	0.24	0.13	0.34	0.22	0.97
	19_20_EpDPE	0.7	0.22	0.38						
	PGD3/EPA	0.52	0.29	0.69						
9	10_NO2_OA	1	0	0	0.35	0.077	0.35	0.053	0.34	0.13
10	Cortisol	1	0.03	0	0.006	0.038	0.69	0.83	0.0045	0.038
