# Supplementary material for: Metabolic alteration in oxylipins and endocannabinoids point to an important role for soluble epoxide hydrolase and inflammation in Alzheimer’s disease—finding from Alzheimer’s Disease Neuroimaging Initiative
Source: Alzheimers Res Ther. 2026 Jan 7;18:21. doi: 10.1186/s13195-025-01939-9 (PMC12857118; doi:10.1186/s13195-025-01939-9)
Supplement: Supplementary file 6 — Supplementary Material 6. [file 13195_2025_1939_MOESM6_ESM.pdf]

Supplemental Table S3.

Cluster	Gender	Anova p value	DunnettTest-MCI to HC	DunnettTest-AD to HC
Cluster 1	male	0.12	0.22	0.073
Cluster 1	female	0.00013	0.92	0.00028
Cluster 2	male	0.00096	0.04	0.00038
Cluster 2	female	0.68	0.66	0.67
Cluster 3	male	0.0056	0.0084	0.0067
Cluster 3	female	0.00018	0.95	0.00042
Cluster 4	male	0.38	0.87	0.63
Cluster 4	female	0.0039	0.0017	0.18
Cluster 5	male	0.0051	0.95	0.011
Cluster 5	female	0.0000027	0.61	0.00018
Cluster 6	male	0.0063	0.44	0.13
Cluster 6	female	0.48	0.39	0.92
Cluster 7	male	0.15	0.74	0.11
Cluster 7	female	0.45	0.88	0.35
Cluster 8	male	0.36	0.26	0.54
Cluster 8	female	0.065	0.037	0.43
Cluster 9	male	0.24	0.34	0.97
Cluster 9	female	0.16	0.13	0.22
Cluster 10	male	0.077	0.053	0.13
Cluster 10	female	0.35	0.35	0.34
Cluster 11	male	0.038	0.83	0.038
Cluster 11	female	0.006	0.69	0.0045
