# Supplementary material for: Metabolic alteration in oxylipins and endocannabinoids point to an important role for soluble epoxide hydrolase and inflammation in Alzheimer’s disease—finding from Alzheimer’s Disease Neuroimaging Initiative
Source: Alzheimers Res Ther. 2026 Jan 7;18:21. doi: 10.1186/s13195-025-01939-9 (PMC12857118; doi:10.1186/s13195-025-01939-9)
Supplement: Supplementary file 7 — Supplementary Material 7. [file 13195_2025_1939_MOESM7_ESM.pdf]

Supplemental Table S4. P values from ANOVA analysis diagnosis group vs metabolites and informative ratios and summations

Metabolite	Group	Group * Sex Int	APOEGrp (0 = e4 negative; 1 = e4 positive)	BMI	Education	Sex	Age
19_20_DiHDPA/19_20_EpDPE	**<.0001**	0.13	0.51	0.0011	0.82	0.97	0.33
14_15_DiHETrE	**<.0001**	**0.048**	0.23	0.01	0.19	0.57	0.38
19_20_DiHDPA	**<.0001**	**0.0016**	0.9	<.0001	0.032	0.0001	0.01
11_12_DiHETrE	**<.0001**	0.055	0.12	0.0003	0.44	0.98	0.2
sum_DiHETrE	**<.0001**	**0.023**	0.15	0.001	0.86	0.53	0.15
8_9_DiHETrE	**0.0001**	**0.049**	0.33	0.06	0.92	0.14	0.15
20_HETE	**0.0002**	0.063	0.059	0.0074	0.7	0.11	0.11
17_18_DiHETE/EPA	**0.0002**	0.82	0.73	0.0049	0.96	0.015	0.62
19_20_DiHDPA/DHA	**0.0002**	0.67	0.88	0.0007	0.86	0.061	0.45
Cortisol	**0.001**	0.43	0.77	0.048	0.47	0.01	0.27
14_15_DiHETE/EPA	**0.0011**	0.87	0.3	0.039	0.31	0.0004	0.084
GDCA/CA	**0.0018**	0.36	0.54	0.47	0.056	0.21	0.12
FA20.3_w6	**0.002**	0.11	0.52	0.22	0.63	<.0001	0.96
GLCA/CA	**0.0038**	0.58	0.83	0.2	0.2	0.24	0.28
UDCA/CA	**0.0042**	0.36	0.66	0.23	0.46	0.61	0.16
HDCA/CA	**0.0044**	0.29	0.97	0.2	0.3	0.17	0.11
17_18_DiHETE	**0.0054**	0.22	0.87	<.0001	0.0049	0.0007	0.057
GDCA	**0.0055**	0.89	0.067	0.17	0.034	0.97	0.99
GLCA	**0.0065**	0.39	0.48	0.68	0.16	0.72	0.63
LCA_3S/CA	**0.0066**	0.61	0.47	0.18	0.34	0.92	0.62
GCDCA/CA	**0.0078**	0.86	0.86	0.16	0.15	0.96	0.24
sum_DiHETE	**0.0094**	0.18	0.96	<.0001	0.0057	0.0016	0.073
1_AG_2_AG	**0.011**	0.11	0.27	0.8	0.28	0.35	0.56
LCA	**0.012**	0.92	0.19	0.11	0.74	0.8	0.17
LPA16.0	**0.021**	0.15	0.92	0.7	0.95	<.0001	0.02
LCA/CA	**0.022**	0.074	0.97	0.31	0.55	0.15	0.22
DCA/CA	**0.023**	**0.017**	0.88	0.87	0.27	0.24	0.026
19_20_EpDPE	**0.028**	0.4	0.76	0.017	0.016	0.0006	0.21
14_15_DiHETE	**0.028**	0.35	0.91	<.0001	0.022	0.041	0.31
GUDCA/CA	**0.029**	0.56	0.6	0.18	0.073	0.13	0.24
LPA22.6	**0.031**	0.32	0.81	0.0036	0.02	<.0001	0.0032
Sphingosine1P18.1	**0.033**	0.52	0.96	0.18	0.019	0.033	0.046
PGD3/EPA	**0.034**	0.76	0.89	0.071	0.34	<.0001	0.61
UDCA	**0.034**	0.75	0.044	0.44	0.54	0.18	0.14
TLCA/CA	**0.036**	0.4	0.82	0.24	0.47	0.051	0.3
GCA/CA	**0.036**	0.89	0.74	0.53	0.31	0.24	0.13
THDCA/CA	**0.038**	0.17	0.92	0.28	0.54	0.61	0.0062
8_HDoHE	**0.042**	0.31	0.72	0.0099	0.0002	<.0001	0.0046

HDCA	**0.042**	0.44	0.18	0.7	0.2	0.44	0.18
TLCA	**0.043**	0.86	0.18	0.8	0.55	0.13	0.29
GLCA/CDCA	**0.045**	0.91	0.25	0.2	0.13	0.14	0.8
TDCA/CA	**0.046**	0.36	0.89	0.45	0.47	0.052	0.13
1_LG_2_LG	0.051	0.11	0.049	0.43	0.011	0.93	0.4
5_HETrE/DGLA	0.055	0.36	0.22	0.82	0.34	0.67	0.58
8_12_iso_iPF2a_VI/AA	0.059	0.069	0.4	0.0009	0.66	0.51	0.2
GDCA/DCA	0.059	0.65	0.43	0.31	0.11	0.82	0.74
FA22.6_w3	0.064	**0.022**	0.84	0.0069	0.006	<.0001	0.079
14,15-DiHETrE/AA	0.067	0.55	0.84	0.11	0.66	0.0013	0.36
15-HETrE/DGLA	0.067	0.084	0.35	0.58	0.49	0.055	0.46
CDCA/CA	0.07	0.3	0.3	0.95	0.94	0.86	0.033
THDCA	0.073	1	0.15	0.34	0.56	0.35	0.11
LPI16.0	0.079	**0.024**	0.9	0.71	0.61	0.0007	0.66
LPA22.5	0.081	0.52	0.86	0.025	0.7	<.0001	0.59
LPA20.5	0.082	0.43	0.33	0.044	0.0085	<.0001	0.011
19_20_EpDPE/DHA	0.082	0.3	0.43	0.81	0.95	0.15	0.78
FA20.4_w6	0.084	0.33	0.35	0.85	0.83	0.0009	0.84
LPA20.3	0.084	0.74	0.85	0.23	0.99	<.0001	0.74
Sphinganine1P18.0	0.087	0.67	0.65	0.34	0.24	0.0002	0.3
DHEA	0.087	0.068	0.073	0.86	0.017	0.077	0.015
5_HEPE	0.088	0.5	0.6	0.13	0.087	<.0001	0.09
LPA20.4	0.088	0.95	0.86	0.4	0.29	0.0095	0.36
16_HDoHE	0.099	0.99	0.89	0.1	1	0.28	0.23
TCDCA/CA	0.1	0.85	0.95	0.21	0.25	0.057	0.05
16-HDoHE/DHA	0.1	0.97	0.85	0.26	0.41	0.75	0.15
TDCA	0.1	0.87	0.24	0.41	0.71	0.18	0.96
GCDCA	0.1	**0.046**	0.19	0.82	0.17	0.098	0.37
LPA16.1	0.1	0.19	0.86	0.017	0.47	<.0001	0.0083
11_12_DiHETrE/14_15_DiHETrE	0.11	0.87	0.55	0.043	0.55	0.76	0.41
14_15_DiHETrE/11_12_DiHETrE	0.11	0.87	0.55	0.043	0.55	0.76	0.41
5_6_DiHETrE	0.11	**0.048**	0.18	0.025	0.25	0.37	0.063
LPE22.6	0.12	0.053	0.92	<.0001	0.013	<.0001	0.001
HCA/HDCA	0.12	0.53	0.98	0.61	0.12	0.39	0.96
LPS18.1	0.12	0.91	0.46	0.14	0.66	0.22	0.35
GCDCA/CDCA	0.12	0.39	0.47	0.21	0.16	0.87	0.54
LPA18.0	0.12	0.37	0.69	0.34	0.77	<.0001	0.88
12_HETE	0.14	0.88	0.5	0.34	0.97	0.048	0.29
(GCA+TCA)/CA	0.15	0.34	0.56	0.82	0.54	0.065	0.079
10_NO2_OA	0.15	0.14	0.34	0.0012	0.77	0.0003	0.044
LPA22.4	0.15	0.7	0.96	0.76	0.066	0.051	0.25
PGD3	0.15	0.95	0.41	0.24	0.045	0.75	0.23
TCA/CA	0.16	0.33	0.56	0.83	0.55	0.06	0.079
GCA/GDCA	0.16	0.29	0.88	0.64	0.13	0.8	0.31
FA18.1_w9	0.16	**0.034**	0.84	0.3	0.69	<.0001	0.066

LPI16.1	0.16	0.38	0.92	0.0005	0.81	<.0001	0.92
8-HDoHE/DHA	0.17	**0.035**	0.58	0.75	0.007	0.65	0.0056
LPS20.4	0.17	0.57	0.54	0.22	0.22	0.28	0.17
sum_HETE	0.17	0.58	0.17	0.34	0.38	0.0011	0.57
12_HEPE	0.17	0.82	0.69	0.011	0.4	0.0015	0.81
FA20.5_w3	0.17	0.82	0.55	0.0035	0.005	<.0001	0.058
12-HEPE/EPA	0.17	0.75	0.62	0.22	0.46	0.22	0.57
14-HDoHE/DHA	0.17	0.81	0.81	0.2	0.55	0.29	0.69
HCA/CA	0.18	0.66	0.78	0.054	0.75	0.73	0.059
TLCA/CDCA	0.18	0.91	0.52	0.32	0.36	0.0046	0.56
cLPA20.4	0.18	0.68	0.77	0.47	0.097	0.14	0.0042
8iso_PGA1/AA	0.19	0.85	0.14	0.46	0.84	0.011	0.81
11b_PGF2a	0.19	0.84	0.26	0.83	<.0001	0.25	0.0094
LCA_3S	0.19	0.66	0.099	0.56	0.36	0.022	0.012
8iso_PGF3a/EPA	0.21	0.63	0.42	0.17	0.65	<.0001	0.093
11b_PGF2a/AA	0.21	0.87	0.18	0.93	0.0014	0.36	0.091
17_HDoHE	0.21	0.89	0.42	0.07	0.5	0.91	0.35
12-HETE/AA	0.21	0.95	0.76	0.27	0.85	0.42	0.22
14_HDoHE	0.22	0.87	0.78	0.026	0.63	0.024	0.97
TCDCA	0.22	0.14	0.23	0.8	0.21	0.53	0.95
FA20.3_w3	0.22	**0.049**	0.45	0.14	0.22	<.0001	0.62
ETAEA	0.23	0.1	0.8	0.0086	0.6	0.44	0.13
FA22.5_w3	0.23	0.094	0.58	0.025	0.12	<.0001	0.17
LPA18.1	0.23	0.075	0.46	0.06	0.37	<.0001	0.22
CA	0.24	0.054	0.46	0.058	0.64	0.12	0.015
LPG16.1	0.24	0.32	0.68	<.0001	0.6	<.0001	0.75
DCA	0.24	0.58	0.23	0.0012	0.21	0.78	0.98
8_12_iso_iPF2a_VI	0.24	**0.045**	0.91	<.0001	0.2	<.0001	0.03
UDCA/CDCA	0.25	0.9	0.59	0.33	0.41	0.18	0.49
LPE20.4	0.25	0.52	0.66	0.0075	0.053	0.33	0.38
9_HEPE	0.26	**0.014**	0.75	0.39	0.096	<.0001	0.1
GCA	0.26	0.22	0.2	0.17	0.44	0.61	0.37
8iso_PGA2/AA	0.27	0.4	0.081	0.18	0.2	<.0001	0.2
11,12-DiHETrE/AA	0.27	0.62	0.98	0.0094	0.81	0.0005	0.34
LCA/CDCA	0.29	0.54	0.26	0.65	0.51	0.091	0.52
sum_HDoHE	0.29	0.81	0.74	0.0053	0.42	0.015	0.41
PGA2	0.29	0.7	0.57	0.013	0.034	0.42	0.048
LTB4	0.3	0.41	0.6	0.64	0.67	0.67	0.014
8iso_PGA1	0.3	0.33	0.23	0.22	0.82	0.36	0.32
5_iPF2a_VI	0.3	**0.012**	0.31	0.01	0.94	0.35	0.59
9_HOTrE	0.31	0.075	0.61	0.008	0.79	0.014	0.23
(TCDCA+GCDCA)/CDCA	0.31	0.82	0.44	0.22	0.27	0.011	0.92
9-HODE/LA	0.32	0.35	0.87	0.07	0.23	0.3	0.66
LPE20.3	0.32	0.33	0.46	0.89	0.67	0.012	0.92
TCDCA/CDCA	0.32	0.82	0.44	0.22	0.27	0.01	0.91

ResolvinE2/EPA	0.32	0.77	0.92	0.14	0.011	0.027	0.21
LPE22.5	0.32	0.47	0.95	0.015	0.84	0.0053	0.089
FA18.2_w6	0.33	**0.0088**	0.87	0.2	0.59	<.0001	0.098
thromboxane_B2	0.33	0.73	0.99	0.55	0.5	0.67	0.067
LPE18.0	0.34	0.67	0.94	<.0001	0.096	0.0017	0.46
LPE16.0	0.35	0.85	0.85	<.0001	0.87	0.0003	0.048
FA18.3_w3	0.36	0.41	0.86	0.25	0.049	0.025	0.41
20-HETE/AA	0.37	0.95	0.82	0.054	0.94	0.03	0.27
PGA2/AA	0.38	0.41	0.54	0.28	0.49	0.0006	0.6
12-HEPE/12-HETE	0.38	0.85	0.29	0.0032	0.2	0.029	0.0024
LPI18.2	0.38	0.29	0.82	0.89	0.13	0.48	<.0001
12_13_EpOME	0.38	0.28	0.67	0.0021	0.051	0.087	0.84
FA18.3_w6	0.39	**0.013**	1	0.58	0.16	<.0001	0.25
PGE3/EPA	0.39	0.28	0.34	0.87	0.29	0.0033	0.21
FA22.5_w6	0.4	0.12	0.47	0.57	0.78	<.0001	0.52
GLCA/LCA	0.4	0.21	0.7	0.32	0.15	0.65	0.79
LPA14.0	0.4	0.41	0.87	0.034	0.82	<.0001	0.26
1_OG_2_OG	0.41	0.29	0.65	0.68	0.15	0.23	0.37
LPG22.4	0.42	0.47	0.14	0.036	0.01	0.83	0.046
12_13_DiHOME	0.42	0.94	0.48	0.0001	0.015	0.0005	0.47
LPG18.0	0.43	0.053	0.3	0.007	0.77	0.8	0.018
LPI18.0	0.43	0.05	0.69	0.11	0.24	<.0001	0.0069
LPI20.4	0.43	**0.043**	0.23	0.19	0.048	0.084	0.5
LPG22.6	0.43	0.67	0.98	0.017	0.0011	0.31	0.098
5-HEPE/5-HETE	0.44	0.65	0.24	0.082	0.016	0.0015	0.018
5_HETE	0.44	0.69	0.32	0.45	0.34	<.0001	0.29
8iso_PGA2	0.45	0.98	0.13	0.006	0.027	0.0014	0.017
ResolvinE2	0.45	0.79	0.92	0.98	0.24	0.56	0.85
5_iPF2a_VI/AA	0.46	**0.0067**	0.18	0.066	0.87	0.13	0.52
9_KODE	0.46	0.067	0.85	0.28	0.28	0.0042	0.21
LPE18.2	0.47	0.076	0.91	0.003	0.14	0.045	0.16
LPE14.0	0.48	0.16	0.98	0.52	0.1	0.023	0.088
TCA	0.48	0.29	0.07	0.17	0.78	0.58	0.78
TDCA/DCA	0.49	0.33	0.86	0.16	0.58	0.057	0.97
11-HETE/AA	0.5	0.59	0.39	0.7	0.48	0.74	0.47
12_HHTrE	0.51	0.71	0.98	0.76	0.39	0.75	0.1
5-HETE/AA	0.51	0.8	0.86	0.67	0.67	0.87	0.24
(TDCA+GDCA)/DCA	0.51	0.38	0.91	0.14	0.93	0.064	0.89
LPS22.6	0.52	0.053	0.58	0.39	0.15	0.49	0.79
15_HETE	0.52	0.86	0.56	0.95	0.062	0.13	0.22
12_13_EpOME/LA	0.52	0.22	0.65	0.011	0.023	0.17	0.76
GUDCA	0.53	0.5	0.15	0.92	0.097	0.93	0.31
Sphingosine1P18.2	0.53	0.36	0.35	<.0001	0.24	<.0001	0.021
LPA18.2	0.54	**0.003**	0.95	0.0062	0.83	<.0001	0.22
FA20.3_w9	0.54	0.44	0.81	0.97	0.44	<.0001	0.39

LPG20.4	0.55	0.97	0.15	0.2	0.082	0.29	0.043
8,9-DiHETrE/AA	0.57	0.62	0.86	0.18	0.95	0.014	0.33
LPG20.3	0.58	0.58	0.13	0.027	0.25	0.32	0.4
15-HETE/AA	0.59	0.56	0.75	0.83	0.12	0.17	0.11
12_13_DiHOME/LA	0.61	0.3	0.6	0.0021	0.0044	0.2	0.87
13-HODE/LA	0.62	0.3	0.54	0.013	0.13	0.69	0.59
LPE20.5	0.62	0.14	0.6	0.0004	0.04	0.0018	0.11
delta17_6_keto_PGF1a/EPA	0.62	0.56	0.56	0.22	0.33	0.054	0.9
GCDCA/GLCA	0.63	0.38	0.66	0.85	0.99	0.088	0.61
5-HEPE/EPA	0.64	0.55	0.79	0.0003	0.015	0.31	0.73
cLPA16.1	0.64	0.95	0.26	0.096	0.8	0.0003	0.12
LPG18.1	0.65	0.11	0.34	0.011	0.81	0.033	0.89
LPG18.2	0.65	0.28	0.29	0.46	0.18	0.31	0.29
GUDCA/UDCA	0.66	0.55	0.69	0.55	0.22	0.35	0.95
AEA	0.67	0.24	0.19	0.63	0.25	0.088	0.73
LTE4	0.67	0.88	0.3	0.72	0.28	0.029	0.73
9_HODE	0.68	0.71	0.76	0.019	0.46	0.0002	0.88
(GLCA+TLCA)/LCA	0.68	0.62	0.94	0.4	0.42	0.47	0.81
LPE22.4	0.69	0.72	0.41	0.61	0.088	0.17	0.038
8iso_PGF3a	0.69	0.84	0.51	0.4	0.04	0.52	0.029
thromboxane_B3	0.7	0.88	0.84	0.093	0.045	0.93	0.93
LPG14.0	0.71	**0.022**	0.7	0.0037	0.49	0.24	0.017
11_HETE	0.72	0.99	0.86	0.59	0.39	0.0051	0.52
LPG16.0	0.73	0.054	0.66	0.015	0.15	0.5	0.12
LPE16.1	0.73	0.25	0.81	0.017	0.82	<.0001	0.94
(TCA+GCA+TDCA+GDCA)/(GUDCA+GLCA+TLCA+TCDCA+GCDCA)	0.73	0.43	0.24	0.39	0.61	0.34	0.47
12_13_DiHOME/12_13_EpOME	0.74	0.15	0.82	0.81	0.7	0.2	0.65
LPI22.4	0.75	0.24	0.38	0.0075	0.23	0.5	0.29
TLCA/LCA	0.77	0.73	0.61	0.32	0.71	0.088	1
delta17_6_keto_PGF1a	0.79	0.52	0.45	0.88	0.82	0.91	0.64
PGE3	0.82	0.24	0.34	0.016	0.32	0.9	0.98
DCA/(LCA+UDCA)	0.82	0.34	0.79	0.073	0.46	0.77	0.16
sum_HODE	0.84	0.62	0.62	0.0075	0.36	<.0001	0.55
LPE18.3	0.86	0.19	0.36	0.0001	0.0048	0.68	0.91
LPE18.1	0.87	0.094	0.49	0.016	0.75	0.74	0.19
(GDCA+GLCA)/(TDCA+TLCA)	0.87	0.1	0.28	0.81	0.23	0.011	0.77
CDCA	0.88	0.53	0.038	0.053	0.62	0.04	0.76
13_HODE	0.89	0.5	0.46	0.0022	0.32	<.0001	0.26
5_HETrE	0.91	0.78	0.42	0.76	0.33	0.0022	0.47
sum_HETrE	0.92	0.9	0.72	0.82	0.71	0.078	0.16
HCA	0.94	0.072	0.17	0.67	0.5	0.079	0.45
17-HDoHE/DHA	0.94	0.13	0.39	0.75	0.51	0.008	0.86
LPG18.3	0.94	0.14	0.038	0.39	0.13	0.91	0.65

Sphingosine1P16.1	0.94	0.46	0.65	<.0001	0.75	0.001	0.0061
LPI18.1	0.95	**0.0056**	0.21	0.64	0.83	0.069	0.57
GDCA/GLCA	0.95	0.72	0.22	0.2	0.27	0.86	0.51
9-HEPE/EPA	0.96	0.31	0.76	0.062	0.0003	0.45	0.66
5,6-DiHETrE/AA	0.96	0.59	0.62	0.044	0.31	0.067	0.065
TDCA/TLCA	0.96	0.91	0.93	0.29	0.84	0.41	0.24
15_HETrE	0.97	0.88	0.76	0.66	0.38	0.15	0.45
cLPA14.0	0.98	0.23	0.79	0.056	0.57	0.38	0.0003
