# Supplementary material for: Metabolic alteration in oxylipins and endocannabinoids point to an important role for soluble epoxide hydrolase and inflammation in Alzheimer’s disease—finding from Alzheimer’s Disease Neuroimaging Initiative
Source: Alzheimers Res Ther. 2026 Jan 7;18:21. doi: 10.1186/s13195-025-01939-9 (PMC12857118; doi:10.1186/s13195-025-01939-9)
Supplement: Supplementary file 8 — Supplementary Material 8. [file 13195_2025_1939_MOESM8_ESM.pdf]

Table S5. Detals of stepwise regression analysis presented in the Figure 4.

e2e3

**Step**	**Parameter**	Action	Sig Prob	Seq SS	**RSquare**	Cp	**p**	**AICc**	**BIC**	
1	10_NO2_OA	Entered	0.0004	7.590558	0.1979	26.775	2	135.453	141.249	( )
2	SC_Age	Entered	0.0007	5.790655	0.3489	13.384	3	125.455	133.024	( )
3	LPG16.1	Entered	0.0071	3.109171	0.4299	7.1193	4	120.002	129.257	( )
4	GCDCA/GLCA	Entered	0.0181	2.166827	0.4864	3.3598	5	116.328	127.178	( )
5	17_18_DiHETE/EPA	Entered	0.0672	1.217431	0.5182	2.1238	6	115.145	127.491	( )
6	5_iPF2a_VI	Entered	0.1745	0.649812	0.5351	2.3965	7	115.717	129.457	( )
7	GUDCA/UDCA	Entered	0.6464	0.074138	0.537	4.1995	8	118.264	133.289	( )
8	5_iPF2a_VI/AA	Entered	0.7297	0.042761	0.5382	6.0858	9	121.032	137.224	( )
9	TDCA/TLCA	Entered	0.8341	0.016022	0.5386	8.0432	10	124.012	141.248	( )
10	cLPA16.1	Entered	0.8679	0.010306	0.5388	10.016	11	127.144	145.291	( )
11	16_HDoHE	Entered	0.9004	0.005962	0.539	12	12	130.43	149.349	( )
12	Best	Specific	.	.	0.4864	3.3598	5	116.328	127.178	(x)

e3e3

Step	Parameter	Action	Sig Prob	Seq SS	RSquare	Cp	p	AICc	BIC	
1	SC_Age	Entered	0	17.31012	0.0807	48.575	2	775.461	786.803	( )
2	PTEDUCAT	Entered	0.0007	6.762858	0.1122	37.667	3	765.93	781.028	( )
3	5-HETE/AA	Entered	0.0064	4.268388	0.1321	31.52	4	760.469	779.311	( )
4	LPI20.4	Entered	0.0237	2.895069	0.1456	27.994	5	757.343	779.915	( )
5	LPE22.5	Entered	0.0062	4.178108	0.165	22.019	6	751.778	778.068	( )
6	19_20_DiHDPA/19_20_I	Entered	0.0079	3.850061	0.183	16.671	7	746.667	776.662	( )
7	5_HETrE/DGLA	Entered	0.0232	2.771374	0.1959	13.381	8	743.491	777.178	( )
8	thromboxane_B3	Entered	0.0443	2.150004	0.2059	11.277	9	741.455	778.821	( )
9	PGD3	Entered	0.0638	1.810891	0.2143	9.8207	10	740.048	781.079	( )
10	5-HEPE/EPA	Entered	0.0465	2.071401	0.224	7.867	11	738.097	782.78	( )
11	LCA	Entered	0.1055	1.359963	0.2303	7.2712	12	737.541	785.863	( )
12	sum_HETrE	Entered	0.3702	0.415966	0.2323	8.4773	13	738.884	790.831	( )
13	17_18_DiHETE/EPA	Entered	0.3898	0.383299	0.234	9.7457	14	740.305	795.863	( )
14	DCA	Entered	0.3411	0.469958	0.2362	10.849	15	741.562	800.717	( )
15	LPE14.0	Entered	0.3376	0.477061	0.2385	11.938	16	742.817	805.555	( )
16	9-HODE/LA	Entered	0.4065	0.357689	0.2401	13.255	17	744.325	810.633	( )
17	Best	Specific	.	.	0.183	16.671	7	746.667	776.662	(x)

e3e4

Step	Parameter	Action	Sig Prob	Seq SS	RSquare	Cp	p	AICc	BIC	
1	SC_Age	Entered	0	18.21808	0.0873	20.699	2	653.827	664.343	( )
2	19_20_DiHDPA	Entered	0.0002	10.27976	0.1365	8.091	3	641.801	655.79	( )
3	LPE16.0	Entered	0.0194	3.90577	0.1553	4.5405	4	638.317	655.761	( )
4	1_AG_2_AG	Entered	0.0428	2.886267	0.1691	2.4388	5	636.222	657.106	( )
5	17_18_DiHETE	Entered	0.3007	0.748793	0.1727	3.3747	6	637.238	661.544	( )
6	14_15_DiHETrE	Entered	0.3198	0.691998	0.176	4.3913	7	638.351	666.062	( )
7	19_20_DiHDPA/DHA	Entered	0.3612	0.582951	0.1788	5.5629	8	639.638	670.736	( )
8	FA18.1_w9	Entered	0.22	1.051112	0.1838	6.0692	9	640.243	674.711	( )
9	PGD3/EPA	Entered	0.4789	0.350334	0.1855	7.5713	10	641.906	679.726	( )
10	TLCA	Entered	0.5066	0.30898	0.187	9.1322	11	643.647	684.801	( )
11	ETAEA	Entered	0.7283	0.084769	0.1874	11.012	12	645.743	690.211	( )
12	GLCA/CDCA	Entered	0.9138	0.008269	0.1874	13	13	647.971	695.736	( )
13	Best	Specific	.	.	0.1553	4.5405	4	638.317	655.761	(x)

e4e4

Step	Parameter	Action	Sig Prob	Seq SS	RSquare	Cp	p	AICc	BIC	
1	ResolvinE2	Entered	0.0015	5.608013	0.1265	42.627	2	171.924	178.626	( )
2	LTE4	Entered	0.0008	5.473274	0.2499	28.285	3	162.418	171.237	( )
3	Cortisol	Entered	0.0051	3.413658	0.3269	20.093	4	156.367	167.241	( )
4	LPI16.1	Entered	0.0288	1.930406	0.3705	16.329	5	153.572	166.435	( )
5	8_HDoHE	Entered	0.0206	2.041779	0.4165	12.233	6	150.145	164.928	( )
6	GDCA	Entered	0.0266	1.766117	0.4564	8.9595	7	147.194	163.827	( )
7	LPI18.2	Entered	0.1024	0.920363	0.4771	8.2115	8	146.765	165.172	( )
8	TDCA/TLCA	Entered	0.0809	1.023271	0.5002	7.1562	9	145.935	166.04	( )
9	LTB4	Entered	0.1629	0.639443	0.5146	7.247	10	146.409	168.129	( )
10	15-HETrE/DGLA	Entered	0.2414	0.446214	0.5247	7.9147	11	147.608	170.859	( )
11	LCA_3S	Entered	0.2006	0.528293	0.5366	8.3373	12	148.556	173.248	( )
12	5,6-DiHETrE/AA	Entered	0.183	0.56566	0.5494	8.6484	13	149.403	175.442	( )
13	20-HETE/AA	Entered	0.2239	0.467177	0.5599	9.2535	14	150.675	177.963	( )
14	GCDCA/CDCA	Entered	0.4253	0.200594	0.5644	10.655	15	153.077	181.511	( )
15	GCDCA	Entered	0.4052	0.219909	0.5694	11.998	16	155.501	184.973	( )
16	GDCA/GLCA	Entered	0.5634	0.106811	0.5718	13.679	17	158.49	188.885	( )
17	Best	Specific	.	.	0.4564	8.9595	7	147.194	163.827	(x)
