# Supplementary material for: Metabolic alteration in oxylipins and endocannabinoids point to an important role for soluble epoxide hydrolase and inflammation in Alzheimer’s disease—finding from Alzheimer’s Disease Neuroimaging Initiative
Source: Alzheimers Res Ther. 2026 Jan 7;18:21. doi: 10.1186/s13195-025-01939-9 (PMC12857118; doi:10.1186/s13195-025-01939-9)
Supplement: Supplementary file 2 — Supplementary Material 2. [file 13195_2025_1939_MOESM2_ESM.pdf]

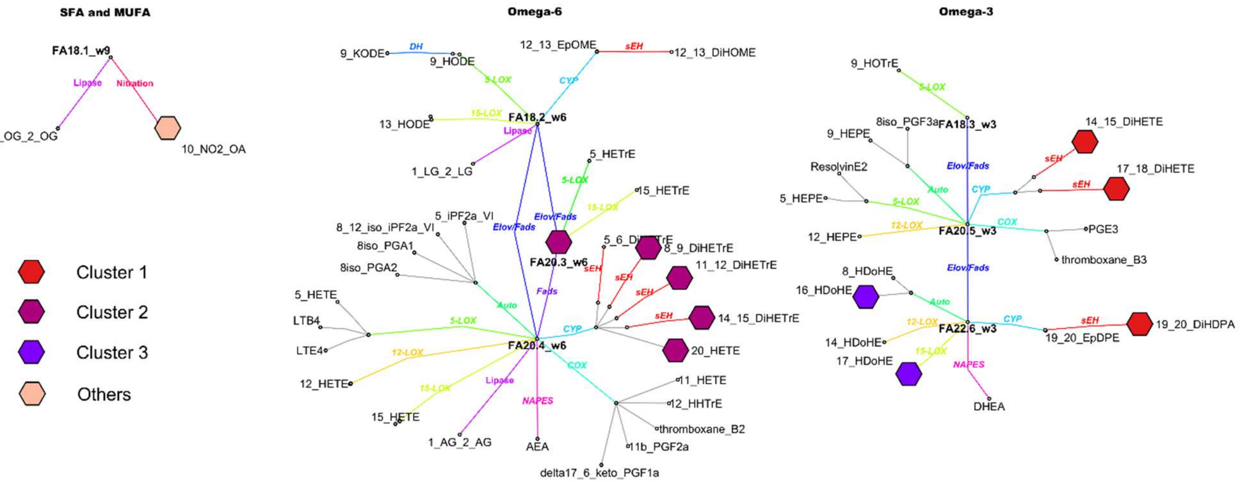

Figure S 2. Metabolites and pathways of oxylipins and endocannabinoids that are implicated in MCI and/or AD in at least one of the sexes. The implicated LPI species and cortisol were not listed in this figure.
